# A randomized placebo-controlled PET study of ketamine´s effect on serotonin_1B_ receptor binding in patients with SSRI-resistant depression

**DOI:** 10.1038/s41398-020-0844-4

**Published:** 2020-06-01

**Authors:** Mikael Tiger, Emma R. Veldman, Carl-Johan Ekman, Christer Halldin, Per Svenningsson, Johan Lundberg

**Affiliations:** 1 Centre for Psychiatry Research, Department of Clinical Neuroscience, Karolinska Institutet and Stockholm Health Care Services, Region Stockholm, Sweden; 2 Department of Clinical Neuroscience, Karolinska Institutet, Stockholm, Sweden

**Keywords:** Depression, Pharmacodynamics, Predictive markers

## Abstract

The glutamate *N*-methyl-d-aspartate receptor antagonist ketamine has a rapid antidepressant effect. Despite large research efforts, ketamine’s mechanism of action in major depressive disorder (MDD) has still not been determined. In rodents, the antidepressant properties of ketamine were found to be dependent on both the α-amino-3-hydroxy-5-methylisoxazole-4-propionic acid (AMPA) and the serotonin (5-HT)_1B_ receptor. Low 5-HT_1B_ receptor binding in limbic brain regions is a replicated finding in MDD. In non-human primates, AMPA-dependent increase in 5-HT_1B_ receptor binding in the ventral striatum (VST) has been demonstrated after ketamine infusion. Thirty selective serotonin reuptake inhibitor-resistant MDD patients were recruited via advertisement and randomized to double-blind monotherapy with 0.5 mg/kg ketamine or placebo infusion. The patients were examined with the 5-HT_1B_ receptor selective radioligand [^11^C]AZ10419369 and positron emission tomography (PET) before and 24–72 h after treatment. 5-HT_1B_ receptor binding did not significantly alter in patients treated with ketamine compared with placebo. An increase in 5-HT_1B_ receptor binding with 16.7 % (*p* = 0.036) was found in the hippocampus after one ketamine treatment. 5-HT_1B_ receptor binding in VST at baseline correlated with MDD symptom ratings (*r* = −0.426, *p* = 0.019) and with reduction of depressive symptoms with ketamine (*r* = −0.644, *p* = 0.002). In conclusion, reduction of depressive symptoms in MDD patients after ketamine treatment is correlated inversely with baseline 5-HT_1B_ receptor binding in VST. Further studies examining the role of 5-HT_1B_ receptors in the antidepressant mechanism of action of ketamine should be conducted, homing in on the 5-HT_1B_ receptor as an MDD treatment response marker.

## Introduction

Subanesthetic dosing of ketamine has brought about a paradigm shift in the treatment of major depressive disorder (MDD), with therapeutic effects within hours rather than weeks^1^. The glutamate hypothesis has so far dominated the research on the antidepressant properties of ketamine^2^. The importance of the glutamate *N*-methyl-d-aspartate (NMDA)-receptor antagonistic properties for the dissociative effects of ketamine has been demonstrated^3^. Diminished antidepressive like response to ketamine has been observed in rodents pretreated with a glutamate α-amino-3-hydroxy-5-methylisoxazole-4-propionic acid (AMPA) receptor antagonist^4^. However, the mechanism of action of ketamine in MDD treatment is still not determined. Without proper understanding of the mode of action, we may fail to utilize the rapid antidepressant actions of ketamine in the development of new drugs for MDD. Compounds that could combine the striking antidepressant effects of ketamine with longer duration of effect, and hopefully without the inherent risk of abuse well known for ketamine, could further revolutionize the field. Furthermore, there is a lack of markers for ketamine treatment response.

Similar to many other effective treatments in psychiatry, ketamine induces a broad spectrum of pharmacodynamic effects. In addition to NMDA receptor antagonism, it also has affinity for opioid, muscarinic, nicotinic, and serotonin (5-HT)_2_ receptors^5^. Most previously characterized antidepressants increase synaptic serotonin concentrations, either through inhibition of reuptake^6^ or degradation of serotonin^7^. The common denominator of serotonergic effect of most antidepressant treatments may also be shared with ketamine^6,8^. Dose-dependent increase of serotonin by ketamine in the prefrontal cortex in rodents is indeed a replicated finding in microdialysis studies^9,10^. Furthermore, serotonin depletion with 4-chloro-DL-phenylalanine abolished the antidepressive-like effects of S-ketamine in rats. The reduced effect on immobility in the forced swim test with S-ketamine in serotonin- depleted rats could be restored with administration of a 5-HT_1B_ receptor agonist^11^.

The 5-HT_1B_ receptor is a potential target for antidepressant treatment based on studies of knockout mice, animal models for depression, MDD case–control studies, antidepressive effects of 5-HT_1B_ receptor ligands, and of the 5-HT_1B_ receptor action of established treatments for depression^12^. In the positron emission tomography (PET) studies of in vivo 5-HT_1B_ receptor binding in MDD published so far, low binding in the hippocampus and in the anterior cingulate cortex (ACC) has been reported both in patients with recurrent MDD^13^ and in patients with MDD and post-traumatic stress disorder (PTSD) comorbidity^14^. Similarly, 5-HT_1B_ receptor binding was low in the ventral striatum (VST)/ventral pallidum in MDD patients with a family history of depression^15^. Conversely, a clinically relevant single dose of the antidepressant escitalopram increased 5-HT_1B_ receptor binding in cortical regions in healthy subjects^16^. Intriguingly, the only published PET study of ketamine effect on 5-HT_1B_ receptors demonstrated an AMPA receptor-dependent increase of 5-HT_1B_ receptor binding in the VST, in non-human primates^17^. VST is a key structure in the reward system, implicated in the anhedonia of MDD^18^. 5-HT_1B_ receptor binding is particularly dense in VST in humans^19^. In line with this, 5-HT_1B_ receptor agonists increase release of the reward behavior regulator dopamine^20,21^. Ketamine increases dopamine levels in VST in rodents^22^. Even though blocking of the MDD treatment effect with a single dose of the opioid receptor antagonist naltrexone has been demonstrated in a pilot study^23^, the role of the reward system in the antidepressant mechanism of action of ketamine has not yet been disentangled.

The primary aim of this study was to measure the effect of a subanesthetic dose of ketamine on 5-HT_1B_ receptor binding with PET and the established radioligand [^11^C]AZ10419369^24^ in selective serotonin reuptake inhibitor (SSRI) treatment-resistant patients with MDD. We hypothesized that ketamine would increase 5-HT_1B_ receptor binding in regions with reported low 5-HT_1B_ receptor levels, such as the hippocampus, ACC, and VST. The secondary aim was to explore correlations between [^11^C]AZ10419369 binding, MDD symptomatology, and treatment response.

## Patients and methods

### Subjects

The study was approved by the Regional Ethical Review Board in Stockholm and by the Radiation safety committee of the Karolinska University Hospital. We strictly adhered to the Helsinki Declaration of 1975 (as revised in 1983). Patients were recruited through internet-based advertisement directing each patient to a home page with the full information regarding participation in the study. After reading the information, the patients could volunteer to participate in the study by filling in study-related forms and self-rating scales for depression: Montgomery-Åsberg Depression Rating Scale (MADRS-S)^25^ and Patient Health Questionnaire (PHQ-9)^26^. Patients fulfilling the study criteria according to self-report were telephone screened by a psychiatrist. Sixty patients were booked for assessment by a psychiatrist performing a standardized clinical interview including Mini International Neuropsychiatric Interview (M.I.N.I.)^27^ and MADRS-rating, after giving written informed consent. Out of a total of 832 volunteers 39 met the criteria for participation and were included in the study. The inclusion criteria were as follows: ongoing major depressive episode according to M.I.N.I., with MADRS ≥ 20, treated for at least 4 weeks with an SSRI in adequate doses without treatment response. The exclusion criteria were as follows: failure to completely fill out the required forms (*n* = 192), any circumstance or condition that could reduce the ability to give informed consent (*n* = 0), not giving informed consent (*n* = 36), not being depressed (*n* = 82), not having received medication for the ongoing depressive episode (*n* = 56), antidepressant treatment response (*n* = 35), bipolar disorder (*n* = 43), psychosis (*n* = 8), neurodevelopmental disorders (*n* = 104), other comorbid disorder as primary diagnoses (*n* = 22), organic brain disorders (*n* = 5), cerebral commotion (*n* = 5), hypertension (*n* = 1), obesity or body weight ≥100 kg (*n* = 47), other significant somatic disorders (*n* = 4), substance abuse (*n* = 31), ongoing fluoxetine treatment (*n* = 25), contraindications to ketamine treatment, inability to perform magnetic resonance imaging (MRI) (*n* = 22), suicidality (rating > 4 on the last MADRS item or reported serious suicidal ideation (*n* = 18)), MADRS < 20 (*n* = 40), pregnancy (*n* = 2), and age <20 years or >80 years (*n* = 12). Three subjects were not included for other reasons. All subjects provided oral and written informed consent after receiving a complete description of the study and before initiation of any study-related events.

The included patients were essentially healthy apart from their MDD, based on medical history, physical examination, electrocardiography, MRI, and standard lab tests. The included patients had an ongoing depressive episode within MDD, with one patient being severely depressed (MADRS > 35) and the others suffering from moderate depression. Mean duration of the current depressive episode was 48 months, with a median duration of 27 months. Twenty of the 39 patients had comorbid conditions according to M.I.N.I. (on average 1.65 comorbid condition per patient in this group) including social phobia (*n* = 9), panic disorder (*n* = 7), agoraphobia (*n* = 6), generalized anxiety disorder (*n* = 4), obsessive compulsive disorder (*n* = 4), antisocial personality disorder (*n* = 2), and PTSD (*n* = 1). Twenty-two of the patients were on antidepressant treatment at the time of inclusion. No patient had responded to the current MDD treatment. Still, the patients were carefully informed about the risks of withdrawing medications. Ongoing pharmacological treatment was washed out for a time corresponding to at least five half-lives of each drug. Nine of the initially included patients were excluded, mostly due to withdrawn consent (*n* = 8). One patient was excluded after testing positive for cocaine and amphetamine in the first drug-screening test. No patient was discontinued after randomization, although one patient declined ketamine continuation treatment due to unpleasant dissociative side effects of the first ketamine infusion. Thirty patients fulfilled the study according to protocol.

### Examination protocol

After washout of medications, when applicable, patients were examined with a baseline PET (PET1). Study treatment was given 1–3 days after PET1. A post-treatment PET2 was normally performed the day after study treatment, although examination within 24–72 h after treatment was according to protocol. For one patient, PET2 was performed the day after a second ketamine infusion, due to radioligand synthesis failure. Patients were assessed by a psychiatrist at the day of PET, with Clinical Global Impression Scales CGI-S (Severity), CGI-I (Improvement) (at PET2)^28^, EuroQol (EQ-)5D^29^, and MADRS^30^. QIDS-SR^31^ was completed by the patient at time of PET and just before first study treatment, and 1, 2, 3, 18, and 24 h after treatment. As side effects of ketamine may entail reduced appetite and disturbed sleep, a shortened version of MADRS omitting the appetite and sleep items (MADRS-short) was used to assess change in depressive symptoms, as preregistered on Aspredicted.org (#17602). Full MADRS was used for baseline measurements. Partial treatment response was defined as 25% reduction of MADRS-short scores, with full response reached when MADRS-short was reduced with 50% or more^32^.

### Study treatment

Treatments were given at Hjärnstimuleringsenheten (a brain stimulation treatment unit), S:t Göran’s hospital, Norra Stockholms psykiatri. The first treatment was randomized, double blind, and placebo-controlled. Patients were randomized to study treatment with racemic ketamine or saline (2 : 1). Randomization was performed with sealed opaque envelopes, each containing one of the study treatment allocations. Before the start of treatment, a nurse not involved in patient assessments opened a new envelope, assigning the patient to the randomized treatment allocation. Active treatment was 0.5 mg/kg racemic ketamine diluted in 100 ml isotonic NaCl solution, given as an intravenous infusion during 40 min. Placebo treatment was an isotonic NaCl solution only. After treatment, patients were observed for 2 h by trained nurses in a hospital setting. Blood pressure, pulse, wakefulness, hallucinations, and anxiety were assessed at baseline and every 15 min during the treatment. After PET2, all 30 patients were offered open-label treatment with racemic ketamine until the last clinical examination in the study. The placebo group was smaller due to the mechanistic focus of the study. Patients and all staff involved in patient assessments and data analysis were blinded to the first study treatment allocation. The week after PET2, the patients were given ketamine infusions twice a week for a total of four treatments. Depressive symptoms were rated with MADRS by medical staff before and directly after each treatment. After completion of the study protocol, each patient was offered an appointment to a psychiatrist for initiation of relapse-preventing treatment.

### Positron emission tomography

PET examinations were performed at the PET center at Karolinska Institutet. During each PET measurement, the subject was placed recumbent with the head in the PET system. Head fixation was achieved with an individual plaster helmet^33^. [^11^C]AZ10419369 was synthesized as previously described^24^. [^11^C]AZ10419369 in a sterile physiological phosphate buffer (pH 7.4) solution was diluted with saline to a volume of 10 ml and then injected as a bolus during 10 s into a cannula inserted into an antecubital vein. The cannula was then immediately flushed with 10 ml saline. Each patient was examined with PET and the radioligand [^11^C]AZ10419369 before (PET1) and after the first study treatment (PET2) (mean injected dose at PET1: 358.3 ± 79.9 MBq, mean injected dose at PET2: 367.5 ± 61.7 MBq). There was no significant difference in injected radioactivity between the first and second PET measurement (*p* = 0.515). The specific radioactivity was high (369.3 ± 156.3 GBq/µmol at PET1 and 388.8 ± 136.2 GBq/µmol at PET2, *p* = 0.475) and the corresponding injected mass of the radioligand was low (0.55 ± 0.44 µg at PET1 and 0.48 ± 0.21 µg at PET2, *p* = 0.346). The ECAT HRRT (High Resolution Research Tomograph, Siemens Molecular Imaging, TN, USA) PET system was used, with a spatial resolution of ~1.5 mm in the center and 2.4 mm at 10 cm off-center full-width at half maximum with the current protocol^34^. Brain radioactivity was measured in a series of consecutive time frames for 95 min. The frame sequence consisted of 38 frames with nine 10 s frames, two 15 s frames, three 20 s frames, four 30 s frames, four 60 s frames, four 180 s frames, and twelve 360 s frames.

### Magnetic resonance imaging

T1-weighted MR images, acquired on a 3T GE MR750 scanner (GE Medical Systems, Milwaukee, WI), were realigned, co-registered with PET images, and segmented into gray matter, white matter, and cerebrospinal fluid using SPM12 (Wellcome Department of Cognitive Neurology, University College London). Inverted co-registration parameters were obtained to transform ROIs from MR to PET space. The preselected regions of interest (ROIs) were hippocampus, VST, ACC, and Dorsal BrainStem (DBS). ROIs were defined using Freesurfer (5.0.0, http://surfer.nmr.mgh.harvard.edu/), except for the DBS ROI. The DBS ROI was defined using [^11^C]AZ10419369 template data in the standard reference space of the Montréal Neurological Institute^35^ and was transformed into individual space using FSL (FSL 5.0, Oxford)^36^.

### PET imaging analysis

Regional binding potential for non-displaceable binding (*BP*_ND_) was calculated using the simplified reference tissue model (SRTM^37^), with the cerebellum as the reference region. The cerebellum ROI was restricted to a smaller region to avoid spill-over from the occipital cortex, cerebrospinal fluid, and cerebellar vermis as described by Matheson et al.^38^. A motion correction algorithm^39^ was applied when subjects showed excessive head movement (see Supplementary Information for further details). Image analysis was performed blinded to treatment allocation.

### Statistical analysis

Statistical analyses were performed in R (version 3.6.1, R Development Core Team, 2019). Differences in regional *BP*_ND_s when corrected for the effect in the placebo group were tested for significance by calculating the group (ketamine vs. placebo) × time (pre vs. post intervention) interaction effect in a repeated-measures analysis of variance (RM ANOVA). For exploratory analysis, regional *BP*_ND_ differences within the treatment group were tested using two-tailed paired *t*-tests. Correlations between regional *BP*_ND_s and MADRS scores were tested for significance using the Pearson’s correlation method. The *α*-level was set to 0.05.

## Results

Thirty patients completed the first study treatment and related examination procedures according to protocol (Table 1). One patient chose to decline further study treatments in the open label phase due to disturbing dissociative side effects. Twenty-nine patients completed study participation including four ketamine treatments with follow up of antidepressant treatment effect.

**Table 1:** Demography.

	Placebo	Ketamine	*p*
Subjects	10	20	
Age, years	37.1	39.2	0.639
Sex	6 F, 4 M	8 F, 12 M	0.442
BMI	25.4	24.8	0.658
MADRS at baseline	30.8	26.3	0.046*

*p < 0.05.

**Fig. 1:**
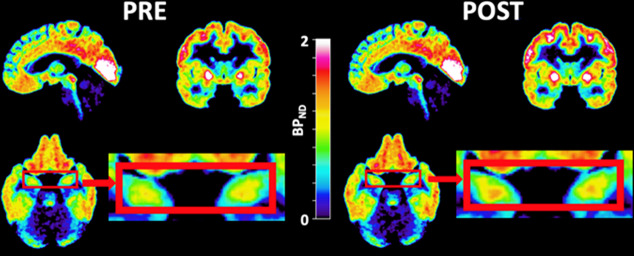
PET images of ketamine treated patients. Average parametric PET images from twenty patients, zooming in on the hippocampi (red boxes, bottom right), before (left) and after (right) ketamine treatment.

In none of the ROIs, a significant difference in *BP*_ND_ change over time between the ketamine and the placebo group was found, as determined by calculating the group by time interaction in RM ANOVA. *BP*_ND_ was increased with 16.7% in the hippocampus (Fig. 1 and Table 2, *p* = 0.036) in response to the first ketamine infusion. There were no significant changes in *BP*_ND_ after ketamine infusion in any other pre-selected ROI. Placebo treatment did not significantly alter [^11^C]AZ10419369 *BP*_ND_ in any region (Supplementary Table). There were no significant differences in total radioactivity, when adjusted for injected dose, in the reference region between PET examinations for the ketamine group (*p* = 0.800), the placebo group (*p* = 0.131), or in changes between groups (*p* = 0.390).

**Table 2:** Mean [^11^C]AZ10419369 *BP*_ND_ in the ketamine group.

Region	Baseline	SD	Treated	SD	*t*-Statistic	*p*-Value
ACC	1.22	0.29	1.34	0.38	−1.755	0.095
DBS	1.31	0.37	1.35	0.47	−0.632	0.535
Hippocampus	0.46	0.16	0.54	0.22	−2.263	0.036*
VST	1.98	0.35	2.09	0.56	−1.162	0.260

[^11^C]AZ10419369 *BP*_ND_ at baseline and after the first ketamine infusion. *ACC* anterior cingulate cortex, *DBS* dorsal brain stem, *VST* ventral striatum. *p < 0.05.

Post-treatment MADRS was significantly lower after active treatment with ketamine compared to placebo (adjusted *p* = 0.019), with MADRS scores decreasing from 26.3 ± 6.58 to 16.00 ± 7.28 after the first ketamine treatment (adjusted *p* < 0.001) compared with that from 30.8 ± 4.92 to 25.00 ± 9.24 with placebo (adjusted *p* = 0.455). After the first treatment, partial response was achieved by 75% treated with ketamine vs. 30% in the placebo group (*p* = 0.045), and 35% of the ketamine-treated patients and 20% of the patients given placebo fulfilled the criterion for response (*p* = 0.675). After four ketamine treatments 84% of the patients initially given ketamine and 90% of the initial placebo group displayed partial ketamine treatment response (*p* = 1) and 74 vs. 70% responded to the ketamine treatment series (*p* = 1). Altogether 72% of the patients in the study responded to the full ketamine treatment, while remission was obtained for 48% of the patients. Patients who received active treatment in the double blinded phase were all convinced that they received ketamine, while four out of ten placebo treated patients thought that they actually were given active treatment. Ketamine treated patients also improved according to QIDS-SR 24 h post infusion (from 17.5 ± 4.1 to 10.9 ± 5.3, *p* < 0.001), and displayed improvements in EQ-5D subjective state of health (from 37.5 ± 17.8 to 57.4 ± 21.0, *p* < 0.001). There were no significant improvements in these self-ratings after placebo treatment. Ketamine-treated patients also displayed improved clinical global impression (CGI-S pretreatment: 4.11 ± 0.48, post treatment: 3.06 ± 1.09, *p* = 0.001; average CGI-I: 2.33) as measured at time of PET. The antidepressant effect of ketamine treatment was rapid, with a significant decrease of QIDS-SR after 1 h (from 17.9 ± 4.1 to 15.3 ± 4.3, adjusted *p* = 0.04) and larger QIDS-SR reduction with ketamine than placebo 18 h after start of infusion (adjusted *p* = 0.01).

**Fig. 2:**
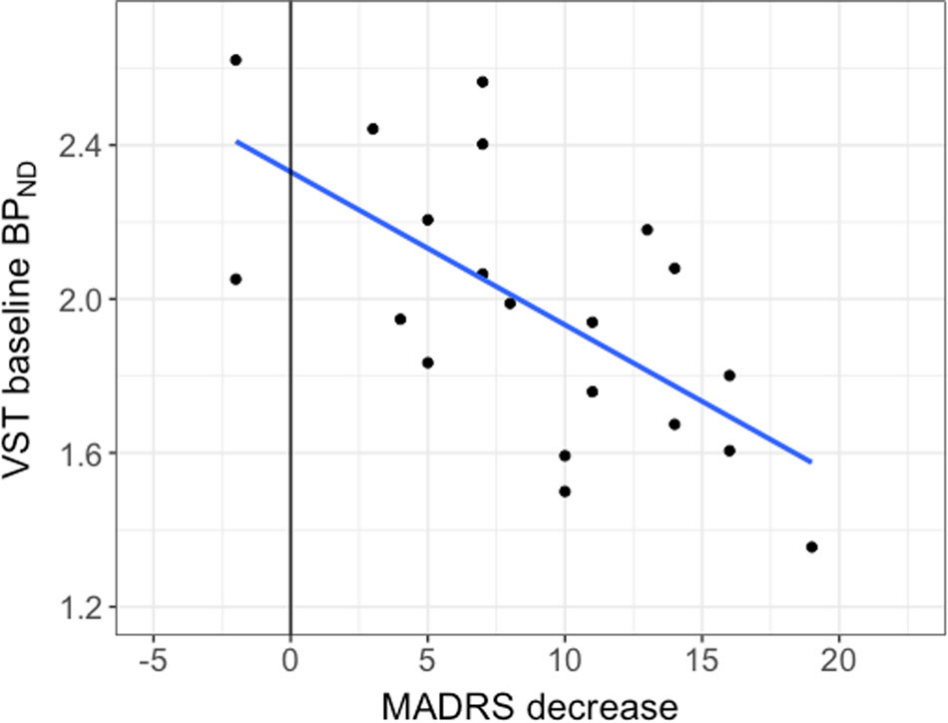
5-HT_1B_ receptor binding in relation to ketamine treatment response. Scatter plot of baseline [^11^C]AZ10419369 *BP*_ND_ in ventral striatum (VST) vs. decrease in MADRS-short after ketamine treatment.

At baseline, *BP*_ND_ in the VST correlated inversely with MADRS (*r* = −0.426, *p* = 0.019), which was not the case in any other selected brain region. In addition, there was an inverse correlation between baseline *BP*_ND_ in VST and change in MADRS-short scores after first study treatment in the ketamine group (*r* = −0.644, *p* = 0.002, Fig. 2). Furthermore, [^11^C]AZ10419369 *BP*_ND_ at baseline in the DBS correlated negatively with change in MADRS-short with ketamine (*r* = −0.510, *p* = 0.022). There were no correlations between baseline *BP*_ND_ and changes in MADRS-short in the placebo group. Changes in *BP*_ND_ with treatment did not correlate with antidepressant effect as measured with MADRS.

## Discussion

In this randomized, placebo-controlled, double-blind PET study, 5-HT_1B_ receptor binding was studied in SSRI resistant MDD patients, before and 24–72 h after ketamine infusion, a time interval chosen for peak antidepressant effect. No significant differences were found in overall [^11^C]AZ10419369 *BP*_ND_ changes pre and post treatment between patients receiving ketamine infusion and the placebo group. In the exploratory analysis, a significant increase in [^11^C]AZ10419369 *BP*_ND_ was found in the hippocampus in patients receiving ketamine treatment. There were no other significant changes in radioligand binding in the preregistered ROIs.

It is not clear why we could not replicate the increased 5-HT_1B_ receptor binding in VST in non-human primates after ketamine infusion^17^. A number of explanations are possible. First, in contrast with the study by Yamanaka et al.^17^, we settled for disentangling the 5-HT_1B_ receptor effect of ketamine by comparing it with placebo treatment, which reduced depressive symptoms in some of the patients. Second, there may be species differences and differences in subject states, where we examined SSRI treatment-resistant MDD patients rather than healthy apes. Third, and perhaps most important, ketamine doses in our study was subanesthetic, whereas in the previous primate 5-HT_1B_ receptor PET study the administered doses were anesthetic^17^.

The hippocampus is a key region in the neurocircuitry of MDD^40,41^. A number of studies have demonstrated ketamine’s effects on hippocampal neurons in rodents^42^. AMPA receptor antagonist pretreatment has blocked ketamine’s reduction of immobility in the forced swim test, while reversing the attenuation of phosphorylation of GluR1 AMPA receptors in the hippocampus induced by ketamine^4^. Optogenetic inactivation of the ventral hippocampus–medial prefrontal cortex pathway has been shown to reverse ketamine’s effect on immobility in the forced swim test^43^. In humans, low 5-HT_1B_ receptor binding in the hippocampus in MDD patients has been reported in previous studies^13,14^. In ketamine-treated patients, [^11^C]AZ10419369 *BP*_ND_ in the hippocampus increased significantly after treatment. The increase in hippocampal [^11^C]AZ10419369 *BP*_ND_ after ketamine may reflect both increased 5-HT_1B_ receptor density and reduced serotonin concentration, as displacement of [^11^C]AZ10419369 binding has been demonstrated after pharmacological challenges expected to increase serotonin levels at least twofold^16,44^. However, with a single dose of 20 mg escitalopram given to healthy human volunteers, there was no radioligand displacement. Furthermore, [^11^C]AZ10419369 *BP*_ND_ has not been shown to correlate with concentrations of serotonin and its metabolite 5-HIAA (5-hydroxyindoleacetic acid) in cerebrospinal fluid^45^. Thus, although a change in serotonin concentration cannot be ruled out, the increase in hippocampal *BP*_ND_ most likely reflects increased 5-HT_1B_ receptor density after ketamine for MDD. Increased 5-HT_1B_ receptor density with ketamine treatment would be in line with the low 5-HT_1B_ receptor binding previously shown in MDD and with the rescue of the antidepressive properties of ketamine by 5-HT_1B_ receptor agonism after serotonin depletion^11^.

In our study, there was a distinct negative correlation between baseline 5-HT_1B_ receptor *BP*_ND_ in the VST and change in MADRS-short associated with ketamine treatment, with larger treatment effects in patients having lower pretreatment 5-HT_1B_ receptor levels. Furthermore, baseline 5-HT_1B_ receptor *BP*_ND_ in the VST correlated inversely with pretreatment MADRS scores. This is in line with the low ventral striatal 5-HT_1B_ receptor *BP*_ND_ previously described in MDD patients^15^. As 5-HT_1B_ receptor stimulation increases dopamine release, low 5-HT_1B_ receptor binding would in theory result in low dopamine levels in VST. VST is a core structure in the reward system and dopamine a major mediator of reward related behavior. Impaired reward reversal learning has indeed been demonstrated in MDD patients, along with reduced striatal activity during unexpected rewards and dopamine dysregulation^46,47^. Ketamine increases dopamine levels in the VST in rodents, even though ketamine-induced dopamine release appears less pronounced in humans^22^. Our results corroborate the 5-HT_1B_ receptor as a biomarker for the depressive state and puts forward 5-HT_1B_ receptor binding as a ketamine treatment response marker.

Even in a well-designed, randomized, placebo-controlled, double-blind, PET study, there are a number of limitations. First, even though this is the largest PET study of ketamine neuroreceptor effects in MDD so far, the sample size is still a limitation, with inherent risk of type II errors, making the nonsignificant differences in 5-HT_1B_ receptor binding in the ketamine group vs. placebo inconclusive. Considering the effect in 5-HT_1B_ receptor binding in the hippocampus seen in our data, a sample size of 110 subjects would be required to detect significant differences between treatment groups with a power of 70%. Second, with the dissociative effects of ketamine, blinding becomes a challenge. The ketamine-treated patients all realized that they received active treatment. However, a surprisingly large part of the placebo-treated patients thought that they had received ketamine as well. This suggest the numerical pre-post differences between ketamine and placebo are most likely not to be driven by unblinding. Third, we studied SSRI treatment-resistant patients. This group may well respond differently to ketamine with regards to the addressed serotonin target the 5-HT_1B_ receptor than non-resistant patients. Thus, we cannot know whether the association between antidepressant effects of ketamine and 5-HT_1B_ receptor binding in the hippocampus can be generalized to the MDD population as a whole. Fourth, the DBS is a region with low signal-to-noise ratio and suboptimal test–retest properties for [^11^C]AZ10419369 in this region with SRTM, the standard reference tissue model for this radioligand^48^. Hence, we also applied wavelet filters (wavelet aided parametric imaging) for DBS data (see supplemental section for further details), but could still not detect any significant changes in 5-HT_1B_ receptor *BP*_ND_ after ketamine infusion (*p* = 0.27).

In conclusion, we found that in patients with SSRI-resistant MDD, reduction of depressive symptoms after ketamine treatment correlated inversely with 5-HT_1B_ receptor binding in the VST at baseline. Even though we found no statistically significant differences in overall 5-HT_1B_ receptor binding changes between ketamin-treated patients and the placebo group, there was an increased 5-HT_1B_ receptor binding in the hippocampus after ketamine treatment. With this study, we suggest a role for the 5-HT_1B_ receptor in the antidepressant mechanism of action of ketamine and we encourage further research that examines 5-HT_1B_ receptor binding and related measurements as response markers for ketamine treatment effect in MDD.

## Supplementary information

Supplementary information

Supplementary table
