# Supplementary material for: A randomized placebo-controlled PET study of ketamine´s effect on serotonin1B receptor binding in patients with SSRI-resistant depression
Source: Transl Psychiatry. 2020 Jun 1;10:159. doi: 10.1038/s41398-020-0844-4 (PMC7261801; doi:10.1038/s41398-020-0844-4)
Supplement: Supplementary file 1 — Supplementary information [file 41398_2020_844_MOESM1_ESM.docx]

Image analysis

*Additional analysis for DBS ROI*

Test-retest reliability for analyzing brainstem ROIs was previously found to be improved (1) when using a wavelet-aided parametric imaging (WAPI) algorithm (2) to create parametric BP_ND_ images. Therefore, BP_ND_ in the dorsal brainstem was also calculated using this method, in addition to SRTM. In this approach a reiterative wavelet-based denoising method was applied before performing a multiple regression analysis based on the non-invasive Logan method (here: t*=18) for calculation of BP_ND_ in each voxel.

*Motion correction*

Motion correction was applied when head movement exceeded (and persisted) a predefined limit of 3 mm in the z-direction. This was the case for 9 PET occasions. In the examinations of four subjects (2 placebo and 2 ketamine treated subjects), movement exceeded a predefined limit in the last three frames. These frames were therefore excluded from the analysis of both PET1 and PET2 data. Another subject showed excess movement in middle frames. These frames were therefore removed. Excluding this subject did not alter the results. Excessive head movement was seen in both ketamine and placebo treated groups, both pre and post intervention.

**References**

1. Nord M, Finnema SJ, Schain M, Halldin C, Farde L. Test-retest reliability of [(11)C]AZ10419369 binding to 5-HT 1B receptors in human brain. Eur J Nucl Med Mol Imaging. 2014;41:301-307.

2. Cselenyi Z, Olsson H, Halldin C, Gulyas B, Farde L. A comparison of recent parametric neuroreceptor mapping approaches based on measurements with the high affinity PET radioligands [11C]FLB 457 and [11C]WAY 100635. NeuroImage. 2006;32:1690-1708.
