# Supplementary material for: A randomized placebo-controlled PET study of ketamine´s effect on serotonin1B receptor binding in patients with SSRI-resistant depression
Source: Transl Psychiatry. 2020 Jun 1;10:159. doi: 10.1038/s41398-020-0844-4 (PMC7261801; doi:10.1038/s41398-020-0844-4)
Supplement: Supplementary file 2 — Supplementary table [file 41398_2020_844_MOESM2_ESM.docx]

Supplementary table

Mean [^11^C]AZ10419369 *BP*_ND_ in the placebo group

| Region | Baseline | SD | Treated | SD | t_statistic | p_value |
| --- | --- | --- | --- | --- | --- | --- |
| ACC | 1.28 | 0.16 | 1.30 | 0.18 | -0.322 | 0.755 |
| DBS | 1.35 | 0.28 | 1.42 | 0.33 | -1.283 | 0.232 |
| Hippocampus | 0.50 | 0.13 | 0.54 | 0.13 | -0.689 | 0.508 |
| VST | 1.95 | 0.32 | 2.00 | 0.35 | -0.746 | 0.475 |

[^11^C]AZ10419369 *BP*_ND_ at baseline and after placebo infusion. ACC= Anterior Cingulate Cortex, DBS= Dorsal brain stem, VST= Ventral striatum. SD= Standard deviation.
